# In Vitro Evaluation of a Stable Monomeric Gold(II) Complex with Hematoporphyrin IX: Cytotoxicity against Tumor and Kidney Cells, Cellular Accumulation, and Induction of Apoptosis

**DOI:** 10.1155/2008/367471

**Published:** 2008-05-15

**Authors:** Georgi Momekov, Dilyan Ferdinandov, Spiro Konstantinov, Sonja Arpadjan, Daniela Tsekova, Galina Gencheva, Panayot R. Bontchev, Margarita Karaivanova

**Affiliations:** ^1^Laboratory of Molecular Pharmacology and Experimental Chemotherapy, Department of Pharmacology, Pharmacotherapy, and Toxicology, Faculty of Pharmacy, Medical University of Sofia, 2 Dunav Street, 1000 Sofia, Bulgaria; ^2^Department of Neurosurgery, “St. Ivan Rilski” University Hospital, Medical University of Sofia, 15 I.E. Geshov Street, 1431 Sofia, Bulgaria; ^3^Department of Analytical Chemistry, Faculty of Chemistry, “St. Kliment Ohridsky” University of Sofia, 1 J. Bourchier Boulevard, 1164 Sofia, Bulgaria

## Abstract

The antineoplastic potential of a stable monomeric Au(II) complex with hematoporphyrin IX (Hp), namely [Au(II)Hp_−2H_.(H_2_O)_2_], was investigated in a panel of tumor cell lines. The complex exhibits strong cytotoxicity, whereby the leukaemia- and lymphoma-derived cell lines are more sensitive, with IC_50_ values comparable to those of the reference anticancer drug cisplatin. In contrast, the solid tumor models are more sensitive to the platinum drug. A comparative assessment of both agents against the human kidney cell line 293T has shown that [Au(II)Hp_−2H_.(H_2_O)_2_] is less cytotoxic. The gold complex induces oligonucleosomal DNA fragmentation in tumour cells following 24-hour treatment and hence its cytotoxic effect is at least partly mediated by induction of apoptotic cell death. A prominent intracellular gold accumulation was detected after treating tumor cells with [Au(II)Hp_−2H_.(H_2_O)_2_] which shows that its putative pharmacological targets are readily accessible after a short incubation period.

## 1. INTRODUCTION

Cisplatin and the structurally related platinum-based drugs represent one of the most important classes of antineoplastic agents, being especially valuable for the treatment of germ cell cancer and a variety of other solid malignancies [1–3]. Despite their important clinical role, however, the platinum-based chemotherapeutics possess relatively low selectivity to malignant cells and hence their application is associated with significant dose-limiting organ toxicities [1]. Beside their unfavorable safety profile, the major limitation in the clinical application of the currently marketed platinum agents is the development of acquired resistance by the tumor cells [2]. Consequently, a significant interest is manifested towards the design and synthesis of cisplatin-dissimilar analogues with modified pharmacological properties capable of bypassing the cellular resistance mechanisms [4–6].

Considering the fact that the thermodynamic stability and kinetic behavior of the metal complexes in biological milieu and hence their biochemical and pharmacological properties depend greatly on the nature of the adduct-forming metal centers, it is well appreciated that a change of the metal ion could alter the antineoplastic activity [6]. Among the nonplatinum metal-based chemotherapeutics much attention has been paid to gold complexes [7]. Well known for their clinical antiarthritic application [8], the gold-based drugs have also attracted interest as potential antineoplastic agents with gold(I)-phosphine derivatives being among the most active in vivo against murine tumor models [9]. Currently, the greatest interest towards development of gold-based chemotherapeutics is focused on the Au(III) compounds which being isoelectronic with platinum(II) share the propensity of forming square planar complexes, analogous to cisplatin [7, 10]. It could be anticipated that similar to Pt(II) compounds, the gold(III) species are capable of binding on DNA and this is the reason for their cytotoxicity. Unlike platinum(II), however, the gold(III) complexes are extremely unstable under physiological conditions which practically preclude the interest towards this class of metal-based drugs. In addition, the gold(III) complexes are highly reactive and are able to oxidize a series of biomolecules such as methionine, glycine, and albumin leading to a quick reduction to gold(I) or even to elemental gold [10–12]. It has been proven that the stability of Au(III) compounds can be augmented by bonding with nitrogen donor-containing bi- and multidentate chelating ligands such as ethylendiamine, cyclam, bipyridine, and so forth, that lower the redox potential of the metal center [10, 13, 14]. Recently, a large number of reports on the preparation, structural characterization, and cytotoxic studies of stable gold(III) complexes and organometallic compounds appeared in the scientific literature [10].

**Figure 1:**
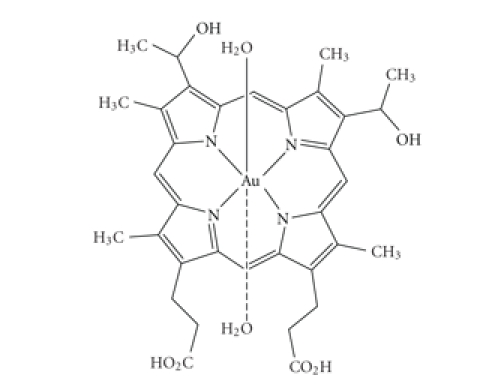
Chemical structure of the tested gold complex [Au(II)Hp_−2H_.(H_2_O)_2_].

With regard to redox stability and kinetic behavior, the search for proper cytotoxic gold(II) complexes is an intriguing and previously unexplored area of anticancer drug design. Nowadays, the Au(II) oxidation state can be considered as a common state in gold chemistry. Despite the large number of stable diamagnetic dinuclear and polynuclear gold(II) complexes, the examples of mononuclear ones are scarce and most of them are with S-containing ligands [15–17]. Recently, the synthesis and structural characterization of a stable monomeric hematoporphyrin Au(II) complex with general formula [Au(II)Hp_−2H_.(H_2_O)_2_] (Figure 1) and distorted octahedral structure has been reported [18]. Au(II) species are stabilized in the complex through coordination via the four nitrogen atoms of the porphyrin macrocycle and the two water molecules are in axial position.

The rationale design for synthesizing porphyrin-based metal complexes as anticancer drugs is based on their selective accumulation within malignant tissue together with participation in augmentation of the cytotoxicity upon light irradiation. Hence, such complexes are expected to behave like hybrid drugs with combined cytotoxic/phototoxic properties [4]. Brunner and coworkers have described large series of planar platinum(II)-porphyrin conjugates whereby the metal centers are coordinated with the porphyrin residues via the pendant functionalities [19–21]. Recently, we have synthesized and characterized three stable octahedral platinum hematoporphyrin complexes in the unusual oxidation state of platinum 3+. In these complexes the hematoporphyrin ligand is coordinated as follows: via four pyrrole N-atoms forming metalloporphyrin type complex; or by asymmetric coordination through two N-atoms from adjacent pyrrole rings forming SAT-type complex; or by the side chains propionic COO^−^groups outside the porphyrin macrocycle. The complexes displayed significant cytotoxic and proapoptotic activities against human tumor cell lines [22].

This study deals with the cytotoxic activity of the newly synthesized stable gold(II) complex Au(II)Hp_−2H_.(H_2_O)_2_ against a spectrum of tumor cell lines. Its effect on the human epidermal kidney cell line 293T has been studied as well in order to estimate the selectivity of cytotoxicity.

## 2. MATERIALS AND METHODS

### 2.1. Chemicals and reagents

RPMI-1640 and DMEM growth media, fetal calf serum, and L-glutamine were purchased from 3. Sigma-Aldrich Co, “St. Louis”, Missouri, USA. (3-(4,5-dimethylthiazol-2-yl)-2,5-diphenyltetrazolium bromide (MTT)- Triton X-100, gold standard, Tris HCl, DMSO, and EDTA were supplied from Merck Co.

The gold(II) complex with hematoporphyrin—[Au(II)Hp_−2H_.(H_2_O)_2_] was synthesized as previously described [18]. The reference anticancer drug cisplatin was purchased from Sigma. Stock solutions of both agents were freshly prepared in DMSO and promptly diluted serially with RPMI-1640 medium to the desired extent. The DMSO concentration never exceeded 1% in the final dilutions obtained.

### 2.2. Cell lines and culture conditions

The T-cell leukaemia SKW-3 (a KE-37 derivative) (DSMZ No.: ACC 53); the nonHodgkin lymphoma DOHH-2 (DSMZ No.: ACC 47); the chronic myeloid leukaemias K-562 (DSMZ No. ACC 10), and LAMA-84 (DSMZ No. ACC 168) as well as the urinary bladder carcinoma-derived 5637 (DSMZ No.: ACC 35) were obtained from DSMZ GmbH (Braunschweig , Germany). The human urinary bladder carcinoma cell line MGH-U1 was supplied by American Type Cell Culture (Rockville, MD, USA). The cells were maintained as suspension type cultures (leukaemias) or as adherent cultures (5637 and MGH-U1) in controlled environment: RPMI-1640 medium, supplemented by 10% heat-inactivated fetal calf serum and 2 mM L-glutamine, at 37°C in a “Heraeus” incubator with 5% CO_2_ humidified atmosphere. In order to keep cells in log phase, the cultures were refed with fresh RPMI-1640 medium two or three times/week.

### 2.3. Cytotoxicity assay

Cell viability was assessed using the standard MTT-dye reduction assay as previously described [23] with minor modifications [24]. Exponentially growing cells were seeded in 96-well flat-bottomed microplates (100 *μ*L/well) at a density of 1 × 10^5^ cells per mL and after 24-hour incubation at 37°C; they were exposed to various concentrations of the tested complexes for 72 hours. For each concentration at least 8 wells were used. After the incubation with the test compounds MTT (3-(4,5-dimethylthiazol-2-yl)-2,5-diphenyltetrazolium bromide (Sigma) solution (10 mg/mL in PBS) was added (10 *μ*L/well). Microplates were further incubated for 4 hours at 37°C and the quantity of formazan product obtained was determined spectrophotometrically using a microprocessor-controlled multiplate reader (Labexim LMR-1) at 580 nm. The cell survival fractions were calculated as percentage of the untreated control (untreated control = 100%). The experimental data were transformed to sigmoidal dose-response curves using nonlinear regression analysis (GraphPad Prizm), which enabled the calculation of the corresponding IC_50_ values.

### 2.4. DNA fragmentation analysis

The characteristic for apoptosis mono- and oligonucleosomal fragmentation of genomic DNA was detected using “Cell Death Detection” ELISA kit (Roche Diagnostics, Germany). The exponentially growing MGH-U1, K-562, HD-MY-Z, and SKW-3 cells were plated in sterile petry dishes and exposed to equipotent concentrations for 24 hours. Cytosolic fractions of 1 × 10^4^ cells per group (treated or untreated) served as antigen source in a sandwich ELISA utilizing primary antihistone antibody-coated microplate and a secondary peroxidase-conjugated anti-DNA antibody. The photometric immunoassay for histone-associated DNA fragments was executed in accordance with the manufacturer's instructions at 405 nm using ELISA reader (Labexim LMR-1). The results were expressed as the oligonucleosome enrichment factor representing the ratio between the absorption in the treated versus the untreated control samples.

### 2.5. Cellular accumulation kinetics

Aliquots of 2 × 10^7^ K-562 cells and HD-MY-Z cells (in 2 mL RPMI 1640) were placed in sterile petry dishes and exposed to different concentrations of the gold complex: (12.5, 25, 50, and 100 *μ*M) for 30 or 60 minutes. After the exposure period, the cells were spun at 2000 rpm for 5 minutes and the drug-containing medium was discarded. The cells were then washed thrice with phosphate buffered saline and aliquots were taken for counting and cell viability determination (trypan blue dye exclusion assay). Thereafter, the cells were digested in 50 *μ*L 10% Triton X100 (EDTA) for 5 minutes, and 950 *μ*L mixture of concentrated hydrochloric acid and concentrated nitric acid (3 : 1) was added in order to allow complete decomposition of the cells, and dissolution of the accumulated gold complex for 1 hour. Following the complete decomposition of the cells, the volume of the samples was adjusted to 1 mL and the gold concentration was determined. The gold concentrations in the sample solutions higher than 0.5 *μ*g/mL were determined using flame atomic absorption spectrometry (FAAS) with an air-acetylene flame (PYE UNICAM SP 1950). Lower gold concentrations were determined by electrothermal AAS using a Perkin-Elmer Zeeman 3030 spectrometer with an HGA-600 graphite furnace. The light source was a hollow cathode lamp for Au. The spectral bandpass was 0.7 nm. Standard uncoated graphite tubes were used as atomizer. Only peak areas were used for quantification. The results were expressed as nmol gold/10^6^ cells.

### 2.6. Data processing and statistics

The cytotoxicity assays were carried out in eight separate experiments, whereas the apoptosis induction evaluation was conducted in quadruplicate. The data processing exploited MS Excel and GraphPad Prizm software for PC. Student's *t*-test was performed with *P* ≤ .05 taken as significance level.

## 3. RESULTS

### 3.1. Cytotoxicity against tumor cell lines

The cytotoxic potential of the novel Au(II) complex was studied in a panel of malignant cell lines, originating from leukaemias, lymphomas, and solid tumors. The results of the chemosensitivity screening program, following 72-hour treatment, are summarized in Table 1. Throughout the screening investigation cisplatin was used as a positive control.

**Figure 2:**
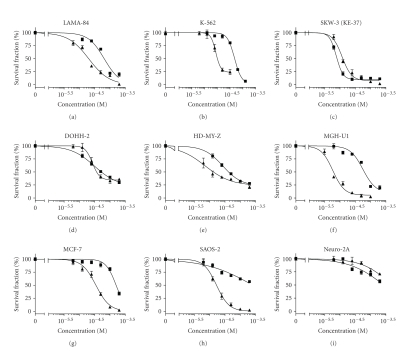
Concentration-response curves of [Au(II)Hp_−2H_.(H_2_O)_2_] (■) and cisplatin (▲) against a panel of tumor cell lines as assessed by the MTT-dye reduction assay after 72-hour exposure. Each data point represents the arithmetic mean ± sd of eight separate experiments.

The cytotoxic effects of the Au(II) complex were evaluated using the concentration-response curves presented in Figure 2. The cellular viability was reduced significantly causing 50% inhibition at micromolar concentrations in the majority of the cell lines tested.

Generally, the leukaemia and lymphoma-derived cell lines were more sensitive to the Au(II) complex and the IC_50_ values obtained were comparable to those of the referent anticancer drug cisplatin. Among them the most responsive tumor model was the T-cell leukaemia SKW-3 (KE-37 derivative) (Table 1, Figure 2). Against this cell line the relative potency of [Au(II)Hp_−2H_.(H_2_O)_2_] even surpassed that of cisplatin. In contrast, the solid-tumor-derived cell lines showed far more pronounced sensitivity to cisplatin as compared to the novel gold(II) complex. The murine neuroblastoma Neuro2A was resistant to both metal complexes.

### 3.2. In vitro cytotoxicity study of [Au(II)Hp_−2H_.(H _2_O) _2_] on human kidney cells in comparison to cisplatin

**Figure 3:**
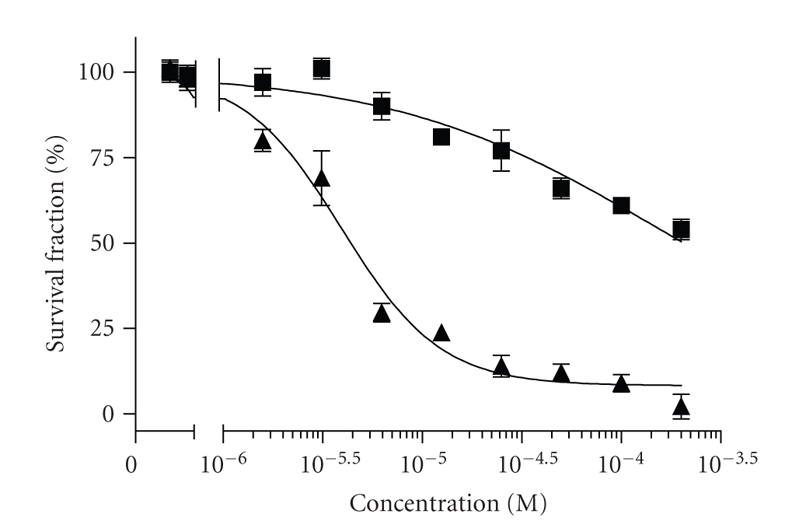
Cytotoxic effects of [Au(II)Hp_−2H_.(H_2_O)_2_] (■) and cisplatin (▲) against the human embryonic kidney cell line 293T as assessed by the MTT-dye reduction assay after 72 hours exposure. Each data point represents the arithmetic mean ± sd of eight separate experiments.

The nephrotoxicity of the novel compound in an in vitro test system versus the established nephrotoxic drug cisplatin was estimated. The human embryonic kidney 293T cells were exposed for 72 hours to either cisplatin or [Au(II)Hp_−2H_.(H_2_O)_2_] and thereafter, their viability was detected with MTT-dye reduction assay (Figure 3). Throughout the tested range of concentrations the gold(II) complex proved to be only marginally cytotoxic and failed to cause 50% reduction of cell viability. In contrast, the referent drug cisplatin exhibited more pronounced cytotoxicity upon kidney cells with an IC_50_ value of 3.87 *μ*M.

### 3.3. Induction of apoptosis following [Au(II)Hp_−2H_.(H _2_O) _2_] treatment

**Figure 4:**
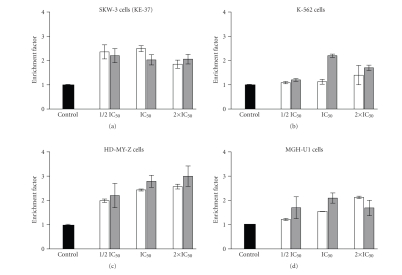
Internucleosomal DNA fragmentation in SKW-3, K-562, HD-MY-Z, and MGH-U1 cells after 24-hour exposure to equipotent concentrations of [Au(II)Hp_−2H_.(H_2_O)_2_] (white columns) or cisplatin (gray columns). The level of DNA fragmentation expressed as the corresponding enrichment factor (ef = 1 in untreated control) was determined using “Cell Death Detection” ELISA (Roche Diagnostics).

Despite their principle modes of action, the majority of anticancer drugs share the distinction of being capable of recruiting the apoptotic cell death signaling pathways in malignant cells. Therefore, the ability of [Au(II)Hp_−2H_.(H_2_O)_2_] to evoke genomic DNA-fragmentation which is a key hallmark of programmed cell death was investigated. For this purpose, the exponentially growing SKW-3, K-562, HD-MY-Z, and MGH-U1 cells were exposed to equieffective concentrations of the gold(II) complex or cisplatin for 24 hours and thereafter, the levels of oligonucleosomal DNA fragmentation were assessed using a commercially available ELISA kit (Figure 4).

The results obtained indicate that [Au(II)Hp_−2H_.(H_2_O)_2_] is a potent apoptosis inductor causing similar level of DNA fragmentation as the referent drug cisplatin if applied at equipotent concentrations. The effect of the gold complex in MGH-U1 and HD-MY-Z was characterized by a straightforward concentration dependence, whereby the proportion of apoptotic cells arise increasing the concentration. In a contrast, the level of oligonucleosomal DNA-fragmentation was found to decrease at the highest concentration as compared to the lower ones in SKW-3. This result could be ascribed to the relative increase of the proportion of necrotic cells undetectable in our experimental setting. The gold complex failed to induce prominent DNA-fragmentation in K-562 cells throughout the concentration range used. The level of apoptosis was significantly higher than the control only after exposure of cells to the highest concentration (twice the IC_50_ value).

### 3.4. Intracellular accumulation of [Au(II)Hp_−2H_.(H _2_O) _2_]

**Figure 5:**
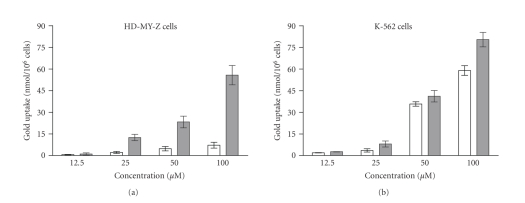
Intracellular accumulation of gold following [Au(II)Hp_−2H_.(H_2_O)_2_] treatment of HD-MY-Z or K-562 cells for 30 minutes (white columns) or 60 minutes (gray columns), means of 4 independent experiments.

The determination of the intracellular levels of gold attained after 30 minutes or 60 minutes of treatment of either K-562 cells or HD-MY-Z with the Au(II) complex is depicted on Figure 5. A prominent time- and concentration-dependent pattern of gold accumulation is evident, whereby the intracellular levels in the K-562 cells are substantially higher as compared to those in HD-MY-Z cells. These data indicate that putative pharmacological targets of the tested compounds are readily accessible after a short incubation period.

## 4. DISCUSSION

To our best knowledge, the present study is the first one addressing the cytotoxic potential of stable monomeric octahedral Au(II) complexes. The antiproliferative activity of the novel compound was evaluated in a wide spectrum of cell lines, representative for some important types of human cancer. The results of the MTT-dye reduction assay unambiguously indicate that [Au(II)Hp_−2H_.(H_2_O)_2_] exerts potent cytotoxic/antiproliferative effect which in some cases is comparable to that of the referent cytotoxic drug cisplatin. Among the cell lines under evaluation the human T-cell leukaemia SKW-3 proved to be the most sensitive to Au(II)-complex treatment, actually the IC_50_ value in these cells was lower than cisplatin. In the other leukaemia models, the relative potency of [Au(II)Hp_−2H_.(H_2_O)_2_] was somewhat lower but more or less comparable to that of cisplatin. Our experimental data indicated that cisplatin was prominently superior against the solid tumor-derived cell lines.

The clinically used platinum drugs as well as the gold antirheumatic agents are characterized by significant nephrotoxicity which is recognized as major dose-limiting factor. Hence, we sought to determine the nephrotoxic potential of [Au(II)Hp_−2H_.(H_2_O)_2_] versus the established nephrotoxin cisplatin. The transformed cell lines appear to be an attractive model among the in vitro test systems used for assessment of nephrotoxicity. They are more versatile than the primary cells on the one hand and retain most of the biochemical features of the normal kidney tissue on the other hand [25]. In the present study, 293T cells were used which have been recently characterized as a suitable model for in vitro assessment of nephrotoxicity [26–28]. The newly synthesized complex [Au(II)Hp_−2H_.(H_2_O)_2_] proves to be far less cytotoxic against kidney cells and in contrast to cisplatin, it fails to induce 50% inhibition of cellular viability. In contrast cisplatin was found to exert prominent cytotoxic effects with the IC_50_ value similar to those obtained in the cancer cell lines.

Another important objective of the present investigation was to determine the intracellular penetration of the gold complex. Although the specific mechanisms of the cytotoxicity of gold species are not fully elucidated, there is a general consensus that they interact with intracellular targets, a feature which is common for most of the anticancer drugs [7, 10]. Thus, the cellular accumulation of the drug appears to be a crucial prerequisite for optimal cytotoxic activity. The novel gold(II) complex is characterised by a significant intracellular accumulation which is more pronounced in the leukemic model K-562 than in HD-MY-Z Hodgkin's lymphoma. A possible explanation is the discrepancy between the cultures type in these cell lines. While the K-562 cells are suspended in the medium, the HD-MY-Z cells tend to attach to the bottom of the cultivation vessel forming monolayers. Conversely, the exposure area in K-562 cells is greater in comparison to that of the semiadherent HD-MY-Z.

In order to elucidate the mechanisms underlying the observed cytotoxicity of [Au(II)Hp_−2H_.(H_2_O)_2_], the level of DNA-fragmentation has been quantified. The induction of programmed cell death appears to be a common feature mediating the cytotoxic effects of anticancer agents and in particular of metal-based drugs [1, 4]. The results reported here confirm the general character of this phenomenon: [Au(II)Hp_−2H_.(H_2_O)_2_] was found to induce apoptotic cell death in SKW-3, MGH-U1, K-562, and HD-MY-Z cells after 24-hour exposure. The low responsiveness of K-562 cells to the proapoptotic effects of [Au(II)Hp_−2H_.(H_2_O)_2_] is the most probable explanation for the discrepancy between its low sensitivity to the gold agent and its excellent intracellular accumulation patterns.

The present data for the Au(II) metalloporphyrin complex are in accordance with the effects established with structurally similar, octahedral Pt(III) complexes which are characterized by a significant cytotoxicity too [22]. The complex [Au(II)Hp_−2H_.(H_2_O)_2_] has an analogous structure with one of these Pt(III) complexes, both having octahedral structure with the metal center being coordinated in the porphyrin ring via the four pyrrolic nitrogens. The juxtaposition of their cytotoxicity shows that while the platinum complex is far more active against the chronic myeloid leukaemia LAMA-84 [18], the Au(II) complex studied exerts superior activity against the T-cell leukaemia SKW3 (KE-37). These data correlate well with the estimated specific inhibiting effect of gold species upon immune cells and T-cells in particular [7].

The selective uptake of porphyrins in malignant tissues/cells is due to complex mechanisms, among them the most important being the LDL-receptor mediated endocytosis of porphyrin/lipoprotein complexes formed in the circulation. Hence, porphyrins are employed as targeting moieties to ensure selective accumulation of cytotoxic agents into the solid tumor microenvironment [19–21]. The novel complex used in the present study demonstrated significant intracellular accumulation presumably mediated by formation of FCS-lipoprotein complexes and subsequent endocytosis. Due to the phototoxic properties of the ligand, a light-borne augmentation of the cytotoxicity of [Au(II)Hp_−2H_.(H_2_O)_2_] could not be ruled out and would be addressed in a further, more detailed evaluation of this compound.

All experimental data presented in this study indicate that [Au(II)Hp_−2H_.(H_2_O)_2_] is a biologically active compound with well-pronounced cytotoxic and proapoptotic properties against malignant cells. As compared to cisplatin, it is less cytotoxic for human kidney cells and this feature may prove to be advantageous.

## Figures and Tables

**Table 1:** IC_50_ values of Au(II)Hp_−2H_.(H_2_O)_2_ and cisplatin against a panel of tumor cell lines assessed after 72-hour exposure (MTT-dye reduction assay).

Cell line	Origin/Cell type	IC_50_(*μ*M)
		Au(II)Hp_−2H_.(H_2_O)_2_	cisplatin
LAMA-84	chronic myeloid leukaemia	65.1	20.3
K-562	chronic myeloid leukaemia	161.2	32.0
SKW-3^(a)^	T-cell leukaemia	7.6	11.7
DOHH-2	nonHodgkin lymphoma	50.1	35.0
HD-MY-Z	Hodgkin's lymphoma	43.1	12.2
MGH-U1^(b)^	urinary bladder cancer	56.4	5.9
MCF-7	breast cancer	166.8	33.4
SAOS-2	osteogenic sarcoma	>200	15.2
Neuro-2a	murine neuroblastoma	>200	>200

^(a)^ KE-37 derivative; ^(b)^ Formerly designated as EJ.
